# Quantification of ovarian lesion and fallopian tube vasculature using optical-resolution photoacoustic microscopy

**DOI:** 10.1038/s41598-022-19778-1

**Published:** 2022-09-23

**Authors:** Xiandong Leng, Sitai Kou, Yixiao Lin, Andrea R. Hagemann, Ian S. Hagemann, Premal H. Thaker, Lindsay M. Kuroki, Carolyn K. McCourt, David G. Mutch, Cary Siegel, Matthew A. Powell, Quing Zhu

**Affiliations:** 1 Biomedical Engineering, Washington University, St. Louis, MO 63130 USA; 2 Department of Obstetrics and Gynecology, Washington University School of Medicine, St. Louis, MO 63110 USA; 3 Department of Pathology and Immunology, Washington University School of Medicine, St. Louis, MO 63110 USA; 4 Department of Radiology, Washington University School of Medicine, St. Louis, MO 63110 USA

**Keywords:** Cancer, Oncology, Engineering, Optics and photonics

## Abstract

The heterogeneity in the pathological and clinical manifestations of ovarian cancer is a major hurdle impeding early and accurate diagnosis. A host of imaging modalities, including Doppler ultrasound, MRI, and CT, have been investigated to improve the assessment of ovarian lesions. We hypothesized that pathologic conditions might affect the ovarian vasculature and that these changes might be detectable by optical-resolution photoacoustic microscopy (OR-PAM). In our previous work, we developed a benchtop OR-PAM and demonstrated it on a limited set of ovarian and fallopian tube specimens. In this study, we collected data from over 50 patients, supporting a more robust statistical analysis. We then developed an efficient custom analysis pipeline for characterizing the vascular features of the samples, including the mean vessel diameter, vascular density, global vascular directionality, local vascular definition, and local vascular tortuosity/branchedness. Phantom studies using carbon fibers showed that our algorithm was accurate within an acceptable error range. Between normal ovaries and normal fallopian tubes, we observed significant differences in five of six extracted vascular features. Further, we showed that distinct subsets of vascular features could distinguish normal ovaries from cystic, fibrous, and malignant ovarian lesions. In addition, a statistically significant difference was found in the mean vascular tortuosity/branchedness values of normal and abnormal tubes. The findings support the proposition that OR-PAM can help distinguish the severity of tubal and ovarian pathologies.

## Introduction

Ovarian cancer is the fifth deadliest cancer among women in the US, and the eighth deadliest worldwide. Over the past decades, despite a lowered incidence rate and improved treatment options, the mortality rate has been reduced only marginally. This is likely due in part to the lack of a reliable screening method and late diagnosis^1,2^. It is reported that only 20–25% of ovarian cancers are diagnosed in an early stage, and most ovarian cancers are diagnosed in stages III and IV^3^. Another major challenge to accurate early diagnosis lies in the heterogeneity of ovarian tumors both histologically and morphologically. According to the dualistic model, epithelial ovarian cancers (EOCs) are categorized as either type I or type II, and each type includes a number of subtypes with distinct histopathological causes and manifestations^4^. In addition, new theories continue to be proposed attempting to explain the origins of different EOC subtypes. For instance, one theory proposes that lesions in the fallopian tube could be the origin of high-grade serous ovarian carcinoma; another finds that endometriosis increases the risk of EOC and is commonly associated with clear cell histology^5–8^. These factors cause considerable uncertainties in identifying the pathologic origins and earliest clinical manifestations of EOC. Various imaging techniques, including transvaginal ultrasound, CT, and MRI, as well as combinations of genetic testing and imaging examinations, have been proposed for early detection of ovarian tumors. However, these methodologies all suffer from low positive predictive values^9,10^. Benign and malignant lesions in the ovary are particularly difficult to distinguish by imaging, although color Doppler ultrasound and contrast-enhanced MRI improve the diagnostic accuracy to a limited extent^11–14^. Alternative imaging techniques are needed to better understand the disease mechanisms and their progression and to more confidently identify ovarian cancers in their early stages.

This study chose to examine the ovarian and tubal vasculature because the altered angiogenesis during the development of ovarian tumors is a clinical target for treatments designed to slow tumor growth^15,16^. Moreover, the angiogenesis in benign and malignant ovarian lesions can be distinguished^17–20^.

In examining vasculature, photoacoustic (PA) imaging offers optically high resolution and high sensitivity to blood vessels^21–23^. In this study, we used OR-PAM to image ovaries and fallopian tubes ex vivo directly after surgical resection to evaluate if we could better determine the risk of malignancy. In our previous report, we demonstrated that our OR-PAM system could resolve blood vessels as small as 5 µm in ovarian and tubal specimens, and subsequently conducted preliminary quantitative analysis using commercial software^24^. For this study, we collected an expanded data set and developed a quantification algorithm to analyze a set of six morphological properties of the vasculature: the mean vessel diameter, vascular density, vascular directionality, vascular definition, tortuosity/branchedness, and PA signal variance. The overall objective of this study is to characterize microscopic features of the vasculature in ovaries and fallopian tubes with various pathologies and to thereby advance our understanding of ovarian neoplasms. To the best of our knowledge, this study is the first one to characterize microscopic vascular features of ovarian lesions and fallopian tubes of various pathologies from a large number of surgical specimens.

## Results

**Figure 1:**
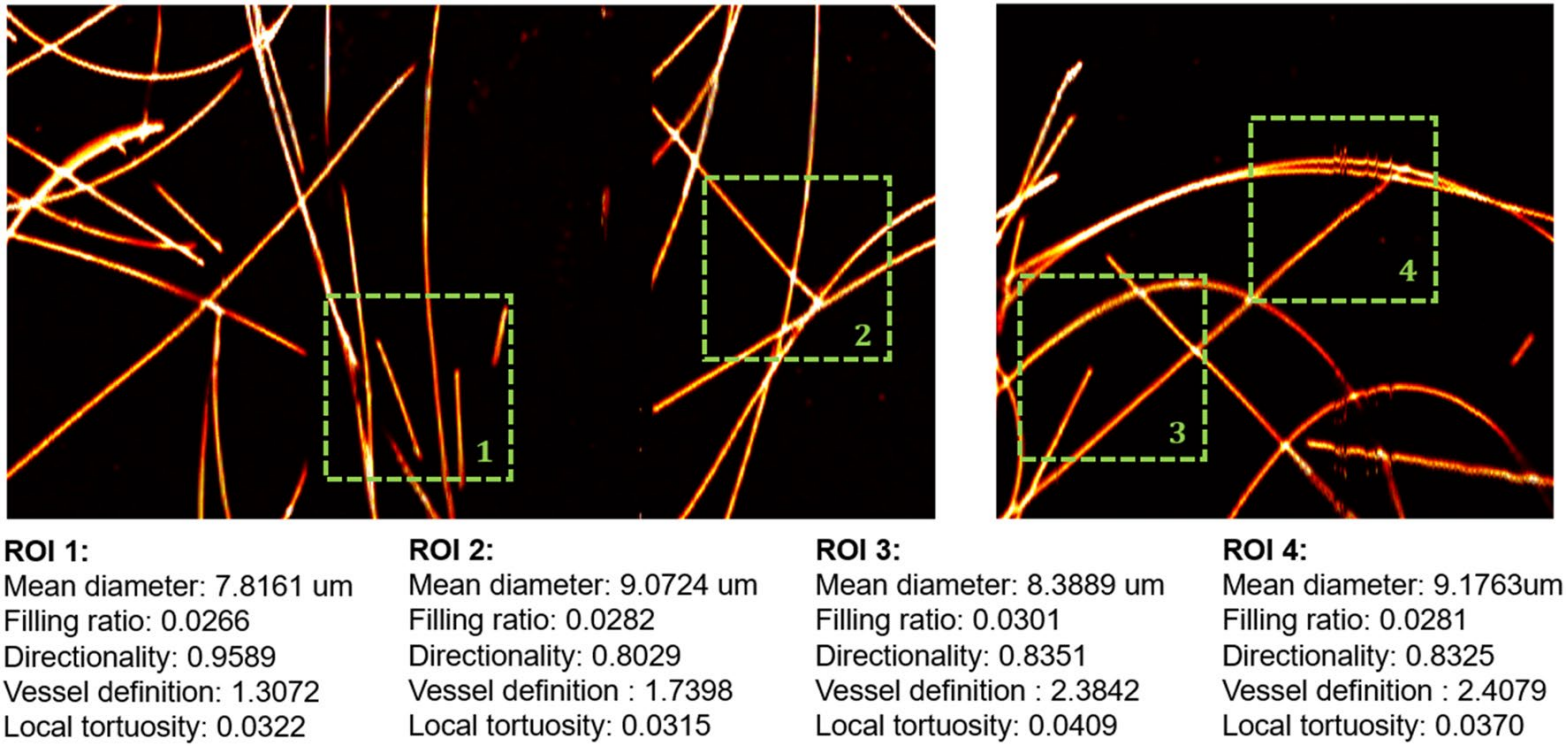
OR-PAM images of carbon fibers. Four ROIs containing carbon fibers with various arranged positions were selected to evaluate the accuracy and consistency of the quantification algorithm.

The vascular quantification scheme extracted the following vascular characteristics: the mean vessel diameter, vascular density, vascular directionality, vascular definition, tortuosity/branchedness, and PA signal variance. Carbon fibers with an average diameter of 7 μm were imaged to validate the quantification scheme. Four regions of interest (ROIs) were selected for testing, as illustrated in Fig. 1. When the carbon fibers were sparse in the images, the estimations of the mean diameter and density were consistent at around 8 μm and 0.028, respectively. The estimated mean diameter was slightly greater than the carbon fiber’s true diameter, suggesting that the estimation scheme would result in overestimation when the blood vessel diameter approaches the resolution limit of the system. Other features were also computed, and the results matched the expectations from visual inspection.

**Figure 2:**
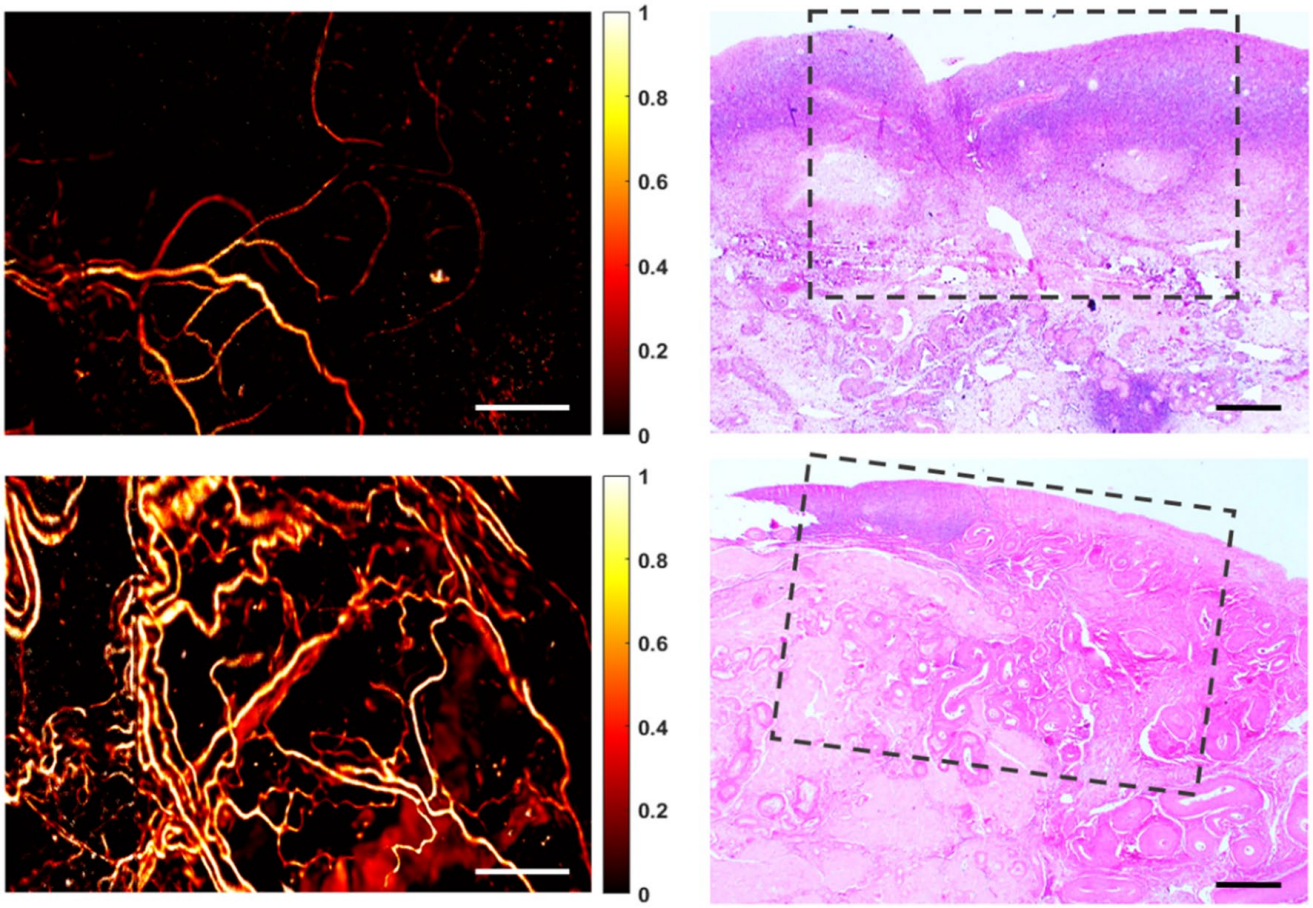
Vasculature of normal ovaries and representative H&E histological images: the ovarian cortex (top) and the venous plexus (bottom). Scale bar: 500 μm. Note that the qualitative comparison of this example and the following examples, Figs. 3, 5 and 6, is based on representative H&Es obtained in the approximate area imaged with the OR-PAM. The need for tissue fixation, processing and sectioning in the pathology laboratory places a limit on our ability to exactly match an H&E section with the area imaged by OR-PAM on fresh tissue.

**Figure 3:**
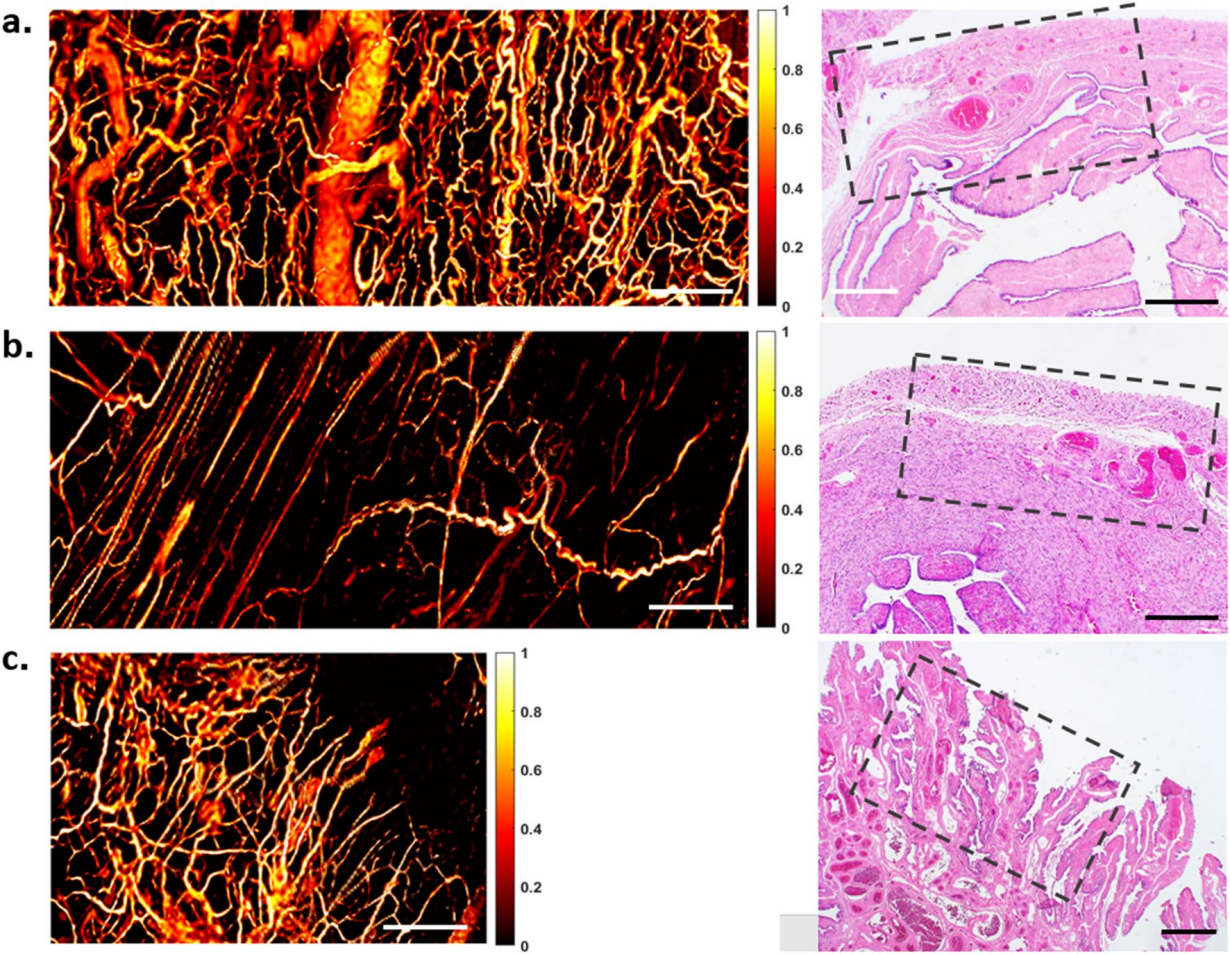
Vasculature of normal fallopian tubes and the representative H&E histological images: (**a**) the isthmus, (**b**) ampulla, and (**c**) fimbriae. Scale bar: 500 μm.

**Figure 4:**
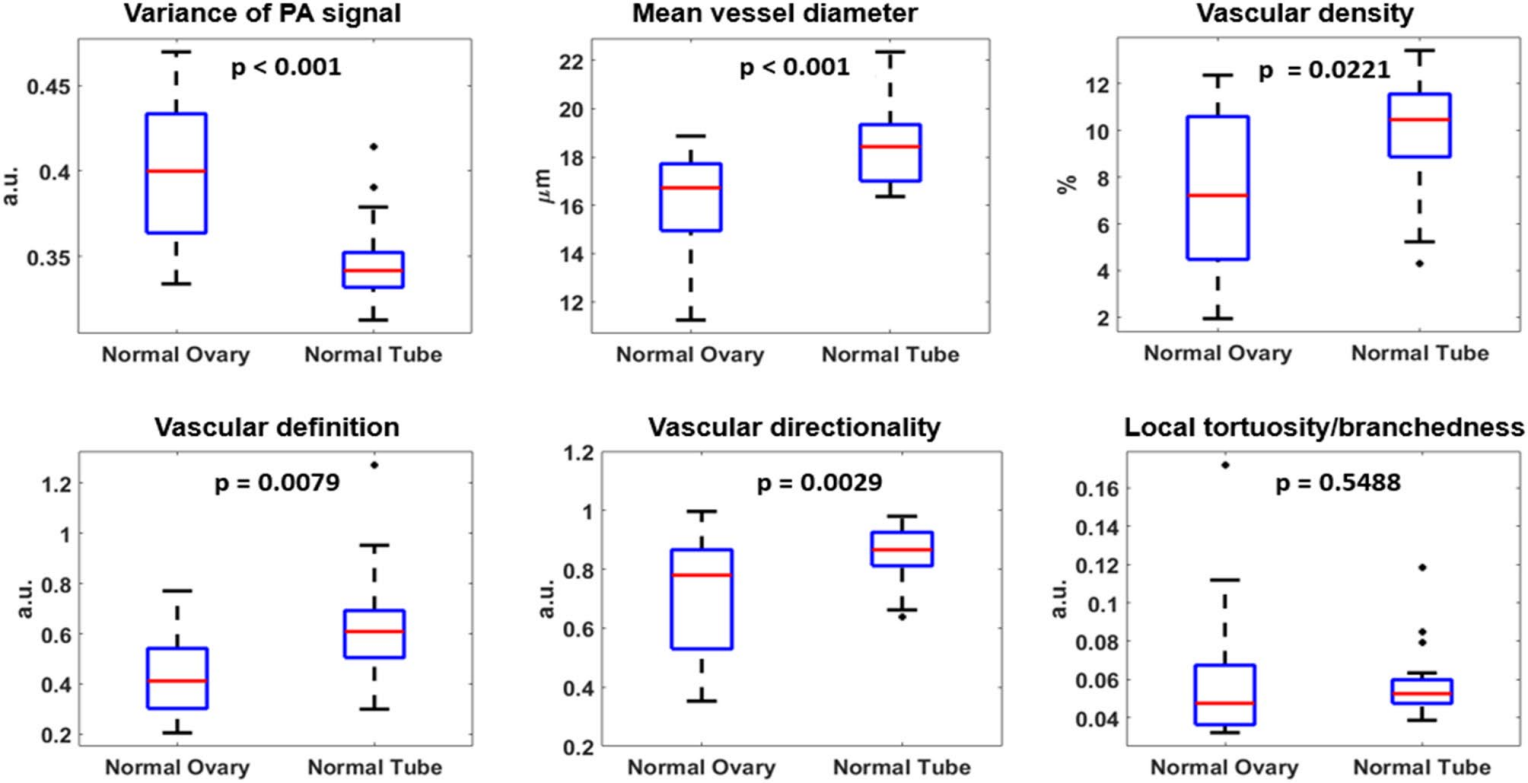
Comparison of morphological vascular feature differences between normal ovaries (n = 10) and normal fallopian tubes (n = 32).

OR-PAM images collected from a cohort of 38 patients were included for analysis. Table 1 provides a summary of the histopathological diagnoses of these samples. The typical vasculature from various anatomical regions of normal ovaries and fallopian tubes is shown in Figs. 2 and 3. In general, distinct vascular features were observed in ovaries and fallopian tubes. Visually obvious, the variation in PA signal intensity was significantly higher in ovaries than fallopian tubes. In addition, our quantification scheme showed a significantly smaller mean vessel diameter and vascular density in normal ovaries compared to normal tubes, $p=0.0008$ and $0.0221$, respectively, which suggested that the ovarian vasculature was narrower and slightly sparser than the tubal vasculature. Moreover, our analysis also indicated quantitatively that the ovarian vasculature had significantly better vascular definition (*p* = $0.0079$), while the tubal vasculature exhibited higher directionality (*p* = $0.0029$), as illustrated in Fig. 4.

**Table 1:** Summary of all different ovarian and tubal pathologies observed in this study, from a cohort of 38 patients with 32 ovarian ROIs and 38 tubal ROIs.

Classification	Description
**Ovarian samples**	
Malignant	High grade serous carcinoma (n = 3), endometrioid carcinoma (n = 1)
Borderline	Borderline ovarian tumor (n = 1)
Benign	Cyst (n = 13) Serous cystadenoma (n = 6), follicular cyst (n = 4), Mucinous cystadenoma (n = 1), cystadenofibroma (n = 2) Fibroma (n = 4) Teratoma (n = 1)
Normal	No histopathological abnormalities (n = 10)

Classification	Description
**Fallopian tube samples**	
Malignant	Metastatic high grade carcinoma of endometrial origin (n = 1)
Benign	Paratubal cyst (n = 2), tubal inflammation (n = 2)
Normal	No histopathological abnormalities (n = 33)

**Figure 5:**
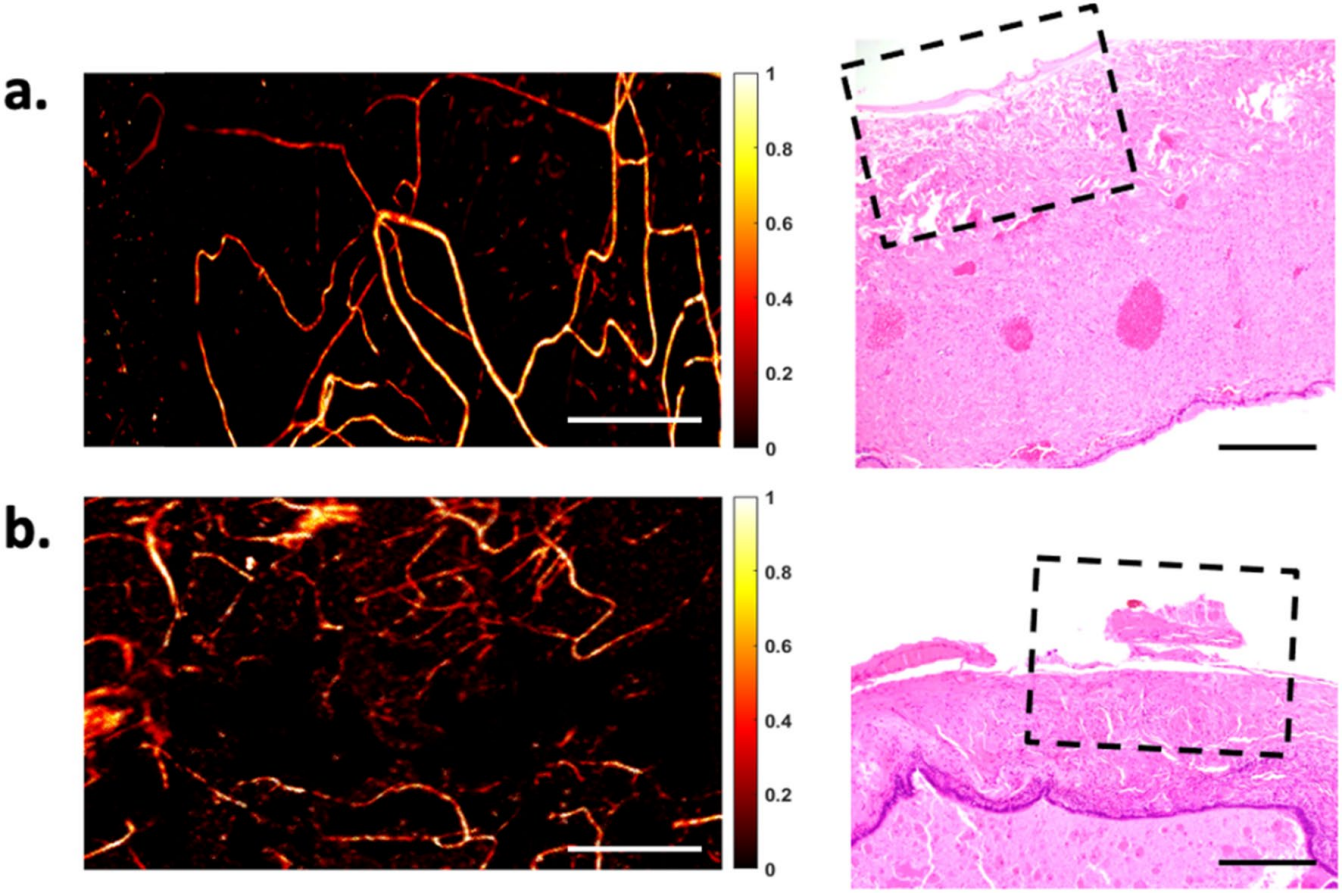
Two different types of benign ovarian lesions and their respective H&E histological images. (**a**) ovary with mucinous cystadenoma, and (**b**) ovary with cystadenofibroma. Scale bar: 500 μm.

**Figure 6:**
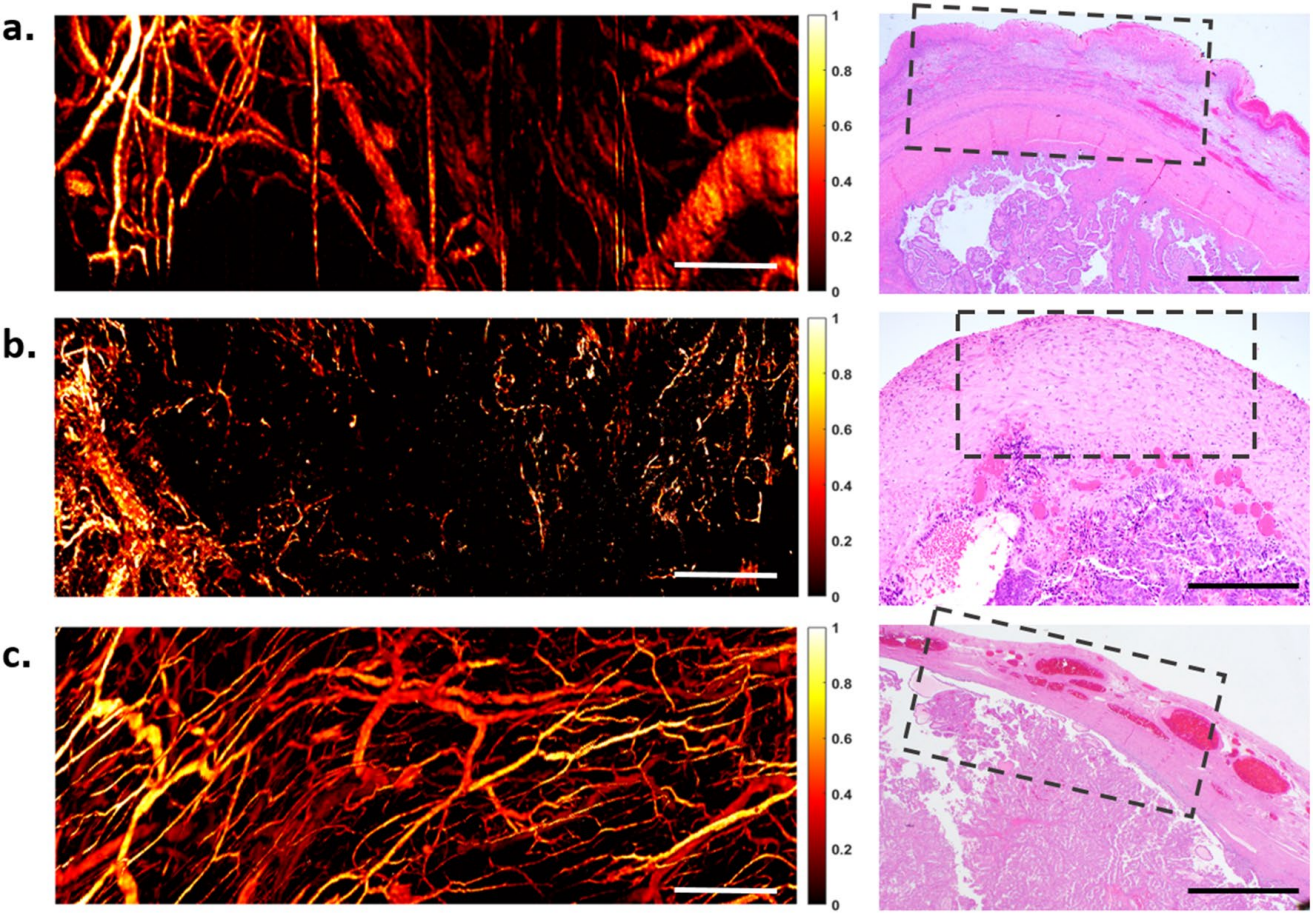
Representative images of ovarian lesions and fallopian tubes with various degrees of malignancies. (**a**) Ovary with borderline tumor, (**b**) ovary with endometroid carcinoma, and (**c**) fallopian tube with metastatic high-grade carcinoma of endometrial origin. Scale bar: 500 μm.

Different varieties of ovarian and fallopian tube pathologies and lesions were examined. Example PAM images of benign and malignant pathologies are shown in Figs. 5 and 6, respectively. They were analyzed using the same set of morphological features and compared to the features’ distributions in normal specimens.

**Figure 7:**
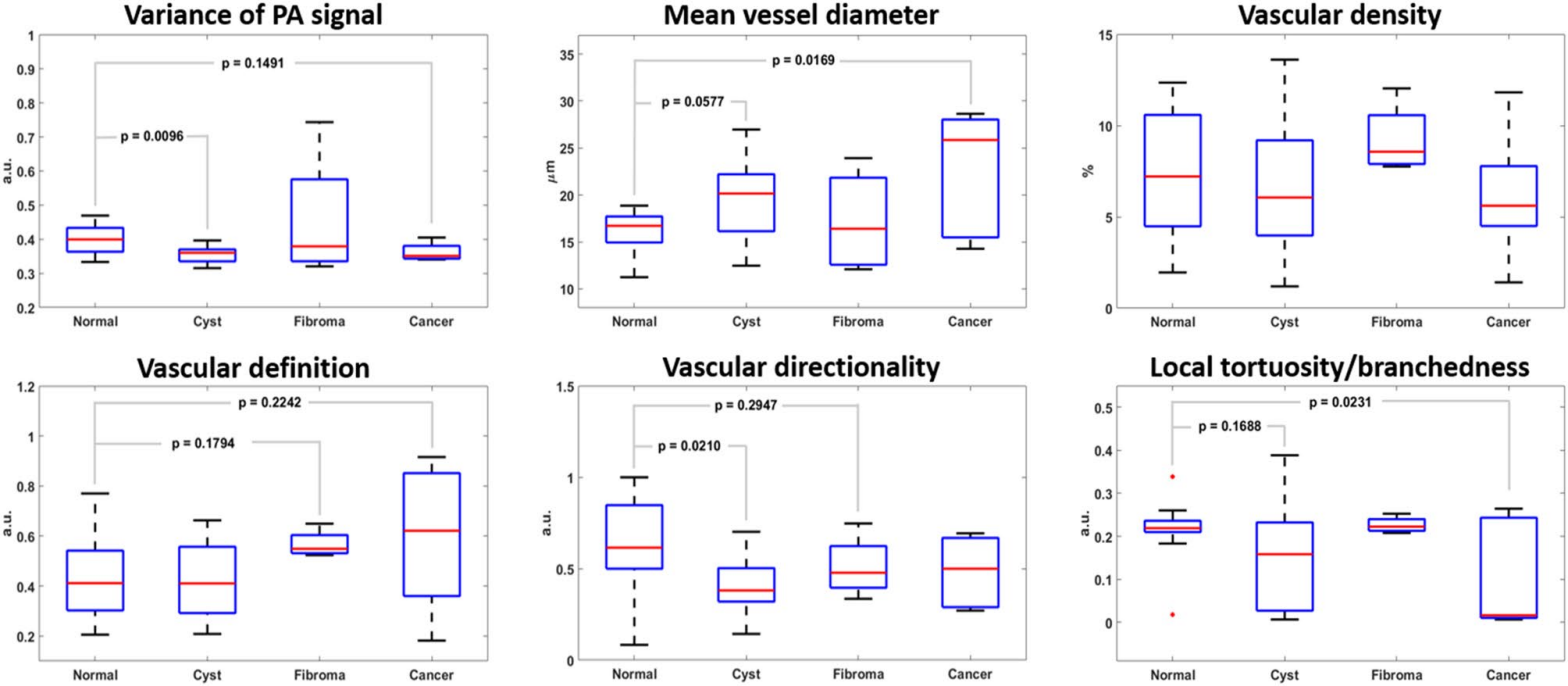
Comparison of morphological vascular feature differences between normal ovaries (n = 10) and ovarian lesions with various degrees of pathology (cyst, n = 13; fibroma, n = 4; malignant, n = 5).

The changes of vascular features in the pathologic ovarian lesions are summarized in Fig. 7. Cystic lesions exhibited significantly lower variation in PA signals, disrupted vascular directionality, and a slight decrease in local tortuosity or branchedness, whereas ovarian lesions with fibroma showed a slightly decreased mean vessel diameter and poorer vascular definition. However, no statistical significance was found in the two features, possibly because there were only four fibromas in the data set. In ovaries with malignant lesions, a significant increase in mean vessel diameter and decrease in local tortuosity were observed, suggesting the loss of fine capillary-level vasculature. A slightly decreased vascular definition and directionality suggested a more disorganized vasculature inside malignant lesions.

**Figure 8:**
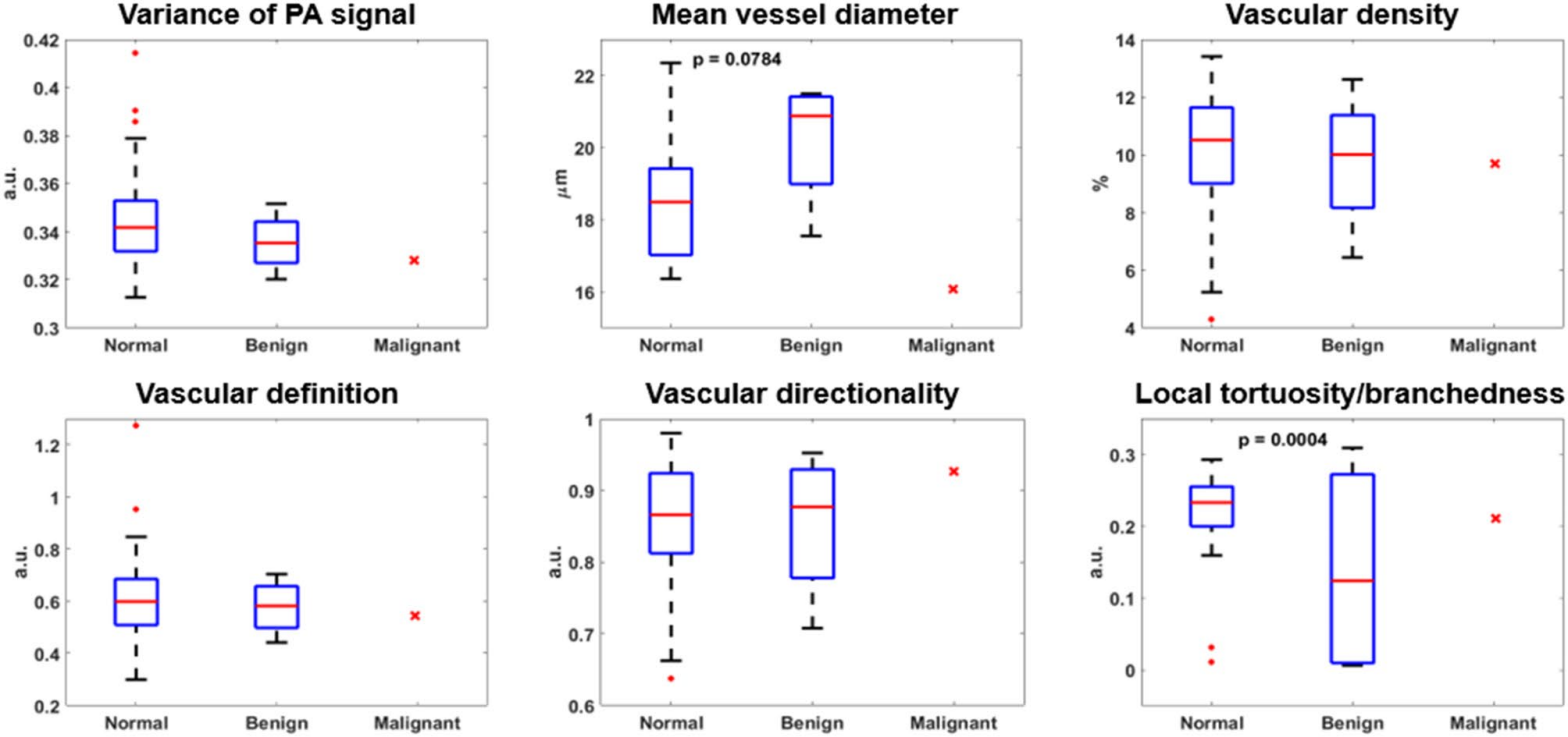
Comparison of morphological vascular features between normal (n = 33) and benign (n = 4) fallopian tubes. A red x marks the results from the single case of malignant tubal lesion, not included in the calculation of *p* values.

Two types of benign fallopian tube pathologies, paratubal cysts and salpingitis, were observed. As shown in Fig. 8, a significantly reduced local vascular tortuosity/branchedness was observed (*p* = 0.0004) in fallopian tubes with benign lesions compared to its normal distribution, alongside a marginal increase in mean vessel diameter (*p* = 0.0784). However, no statistical significance was found for other vascular features. Only one fallopian tube in our data set was diagnosed as malignant, i.e., metastatic high-grade serous carcinoma of endometrial origin. Curiously, in this one case, the trend in vascular changes found in benign pathologies was not observed. The mean vessel diameter was even lower than the normal vessel diameter, while the local tortuosity lay in the normal range.

**Figure 9:**
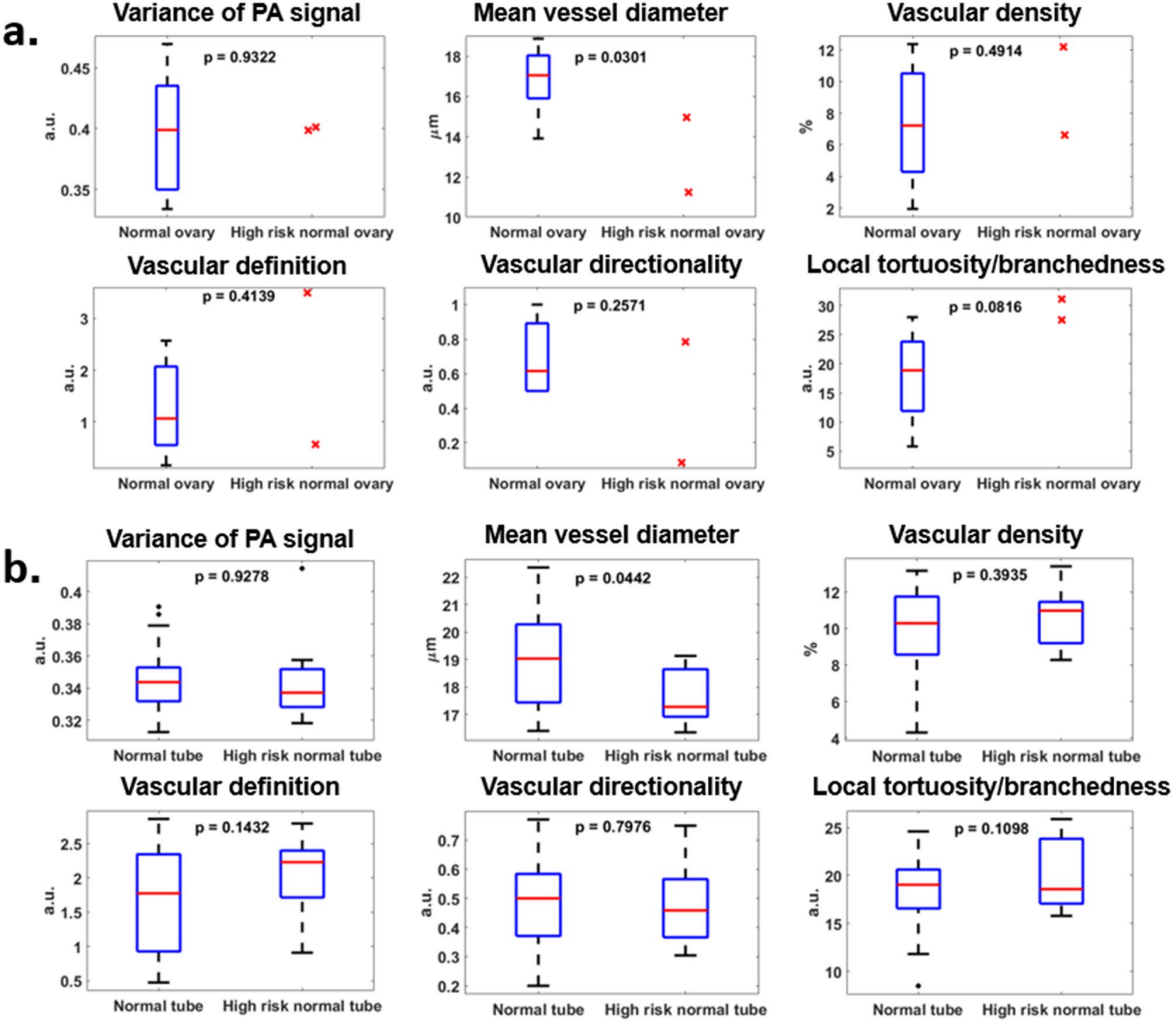
Comparison of morphological vascular feature differences between normal specimens with and without high-risk genetic indicators for ovarian cancers. (**a**) Among ovary specimens, 2 out of 10 cases were high-risk. (**b**) Among tube specimens, 11 out of 32 cases were high-risk.

The comparison of vascular features between high-risk normal specimens from genetic screening and other normal specimens is illustrated in Fig. 9. Due to the limited number of high-risk cases, especially high-risk normal ovaries, robust statistics could not be obtained. However, based on the cases in our data set, no statistically significant difference was observed in most vascular features between normal ovaries (tubes) and high-risk ovaries (tubes). This finding was expected because no histopathological abnormalities were found in these high-risk cases. Curiously, in high-risk ovaries, a slight narrowing of mean vessel diameter was observed, together with a slight increase of local vascular tortuosity/branchedness. In high-risk fallopian tubes, the same change in these two vascular features was observed. Additionally, a slight loss of vascular definition was also found in high-risk fallopian tubes.

## Discussion

In our previous study, we demonstrated the feasibility and suitability of imaging the vascular details of ovarian and tubal excisions using our OR-PAM system^24^. With rudimentary statistical analysis using commercial software, we confirmed our hypothesis that both benign and malignant lesions in the ovary and fallopian tube show alterations in the native angiogenesis. We found that the change in vascular characteristics can be extracted using a quantitative method to provide a better understanding of different ovarian lesion pathologies. Here, we have acquired an expanded data set with a total of 31 ovarian lesion images and 38 fallopian tube images from samples with normal, benign cystic lesions and fibroma, and malignant pathologies. This data set allows us to extract the distributions of vascular features more reliably and compare the changes associated with these common types of ovarian lesions. There are dozens of possible ovarian lesions spanning benign, borderline and malignant categories. Due to limited case numbers in the present study, we cannot obtain more detailed classification based on pathology. We will keep collecting data from ovarian samples with different pathologies and investigate if OR-PAM can indeed distinguish ovarian lesions of various pathologies. Another excellent imaging tool to map ovarian surface vasculature is Optical Coherence Tomography (OCT) angiography, which uses correlation-based algorithms to extract subsurface microcirculation of blood vessel networks^25^. OCT angiography may also have a significant role in assessing ovarian surface blood flow in in vivo studies.

Compared to the commercial program previously used, which was designed to trace filament-like structures in noisy 3D data and required hours of processing on a GPU, the vascular characterization algorithm developed for this study is more efficient and produces output features that have definitive physical correlations with vascular characteristics. The phantom studies on carbon fibers suggest that the algorithm slightly overestimates the mean diameter when the vessels approach the system’s resolution limit or when noise and image artifacts are prominent. Nevertheless, because the typical diameters of ovarian/tubal vessels exceed that of carbon fibers, and there is usually a range of vessel diameters in one single image, the algorithm is expected to produce reasonably accurate estimations. The quantification of other vascular features matches our expectations based on the phantom studies. In addition, to extract more complete vascular information from each OR-PAM image, we selected a combination of global and local features: of the six features, vessel diameter, density, and tortuosity/ branchedness have been indicated as important markers when assessing vascular health associated with various types of cancers and surgical procedures on blood vessels^26–29^, while the rest are quantifications of features unique to OR-PAM based on our observations of the data set.

In terms of system performance, our current OR-PAM system takes 5 min to image a $3\mathrm{ mm}\times 6\mathrm{ mm}$ region. Currently, only 1–2 regions from each ovary or fallopian tube specimen were imaged due to time constraints. We observed that the vasculature in different anatomical regions of the ovary and the fallopian tube sometimes appeared slightly different. Therefore, the field of view of our system can be increased to reveal the vascular variability of larger and different anatomical regions from the same specimen. In addition, to further mitigate the overestimation of the mean vessel diameter and the vascular density, we can improve the axial resolution by upgrading the photoacoustic detection mechanism of our system.

Our validation confirms that OR-PAM identifies vascular structures. A limitation of our method, as we work towards further correlation between OR-PAM and histologic findings, is that it is difficult to obtain an H&E section of the precise area that was imaged by OR-PAM due to the lack of fiducial marking. Moreover, tissue size and shape are altered by standard pathologic handing including opening cystic masses, fixing specimens in formalin, and sectioning them for histology. This limitation does not affect the statistical analysis and conclusions based on OR-PAM.

During the analysis of vascular characteristics, we first observed that the vasculature in the ovary and the fallopian tube is visibly different, which was confirmed by our statistical comparison of different vascular features in normal ovaries and tubes. We then found that some quantitative vascular features were different between pathologic and normal samples with statistical significance. Further, we observed that the vasculature in cystic, fibromatous, and cancerous ovarian samples deviates from its normal conditions in distinct sets of vascular features. Therefore, with a sufficient large data set, we not only can potentially identify abnormalities in the ovary, but also possibly predict the histologic diagnosis based on a combined set of quantitative vascular features from PA images. Due to the limited number of benign and malignant fallopian tube samples in the current data set, the changes in vascular characteristics associated with tubal pathologies remained inconclusive, although the difference in local vessel tortuosity/branchedness has shown statistical significance. There is only one malignant fallopian tube in this reported data set, and its change in vasculature is observed to be opposite to that in malignant ovaries. We will continue to collect ovarian tissue specimens and verify this observation in our ongoing study.

Finally, we compared high-risk normal specimens with other normal specimens and observed a slight decrease in vessel diameter and an increase in local tortuosity/branchedness in the microvasculature in both high-risk ovaries and fallopian tubes. Although we could not show robust statistical differences due to the limited size of our data set, these two vascular features can potentially provide some insight into the clinical manifestations of early ovarian malignancies. More specimens carrying genetic risk factors, within which early malignancies are rarely found, are needed to validate this hypothesis and this is our ongoing work.

## Conclusion

For the first time, we report a microscopic vascular characterization of ovarian lesions and fallopian tubes of normal, benign cystic lesion and fibroma, and malignant pathologies based on a large number of surgical specimens. We develop an efficient analysis scheme which quantitatively extracts morphological vascular features, including the mean vessel diameter, vascular density, global vascular directionality, local vascular definition, and local vascular tortuosity/branchedness. Our analysis shows that normal ovaries and normal fallopian tubes have distinct vascular morphologies. Further, we show that distinct subsets of vascular features could distinguish normal ovaries from cystic, fibromatous, and malignant ovarian lesions. In addition, a statistically significant difference was found in the mean vascular tortuosity/branchedness between normal and abnormal tubes. Our study demonstrates the potential of OR-PAM for distinguishing various common ovarian and tubal pathologies by analyzing the sample’s surface vascular morphology. Modifications on the current OR-PAM system to achieve a faster scanning speed and a larger field of view will provide more accurate visualization and diagnosis of the samples being imaged and better account for variability within and between samples. Microscopic vascular analysis of a larger set of high-risk normal samples could potentially add to the current understanding of how ovarian malignancies develop at their early stages.

## Methods

### Optical resolution photoacoustic microscope (OR-PAM) system configuration

The vasculature of ovarian lesions and fallopian tubes was imaged ex vivo with an in-house OR-PAM system, described in reference 24. The regions of interest (ROIs) were transversely raster-scanned with a step size of 3 μm. Experimentally, the system achieved a lateral resolution of ~ 7 μm. Each imaging session covered a total scanning area of 3 mm ×6 mm, corresponding to 2000 B scans with 1000 A lines in each B scan, and took 5 min to complete.

### Ovarian and fallopian tube specimens

This study was approved by the Institutional Review Board of the Washington University School of Medicine (WUSM). Informed consent was obtained from all patients. All specimens were imaged immediately after surgical resection and returned to the Pathology Department within 1 h for routine standard-of-care processing. WUSM pathologists provided anatomical guidance on the orientation of the ovarian lesions and fallopian tubes, and the lesion location if applicable. The surgical resection could include either one or both ovaries or adnexal masses and fallopian tubes. The specimens were cleaned with deionized water to remove surface blood, and covered with ultrasound gel for better photoacoustic signal coupling. One to three images were obtained from the surface of each ovarian specimen and near the fimbriated end of each tubal specimen, based on the specimen’s size.

### Photoacoustic image pre-processing

The volumetric raw photoacoustic data were stored in a binary file. All image reconstruction was performed with MATLAB 2019R. Hilbert transform was applied to each A line to extract the photoacoustic signal envelope, then, a dynamic range of 33 dB was applied for noise reduction. Each B scan consisted of 1000 A lines, and the even-numbered B scans were flipped and laterally translated to align with the odd-numbered B scans. Each C scan contained 2000 B scans, which corresponded to a volume of 6 mm (length)$\times$ 3 mm (width)$\times$ 0.986 mm (depth). The maximum intensity projection was calculated for each C scan to obtain the specimen’s 2D vasculature map.

### Vasculature characterization

**Figure 10:**
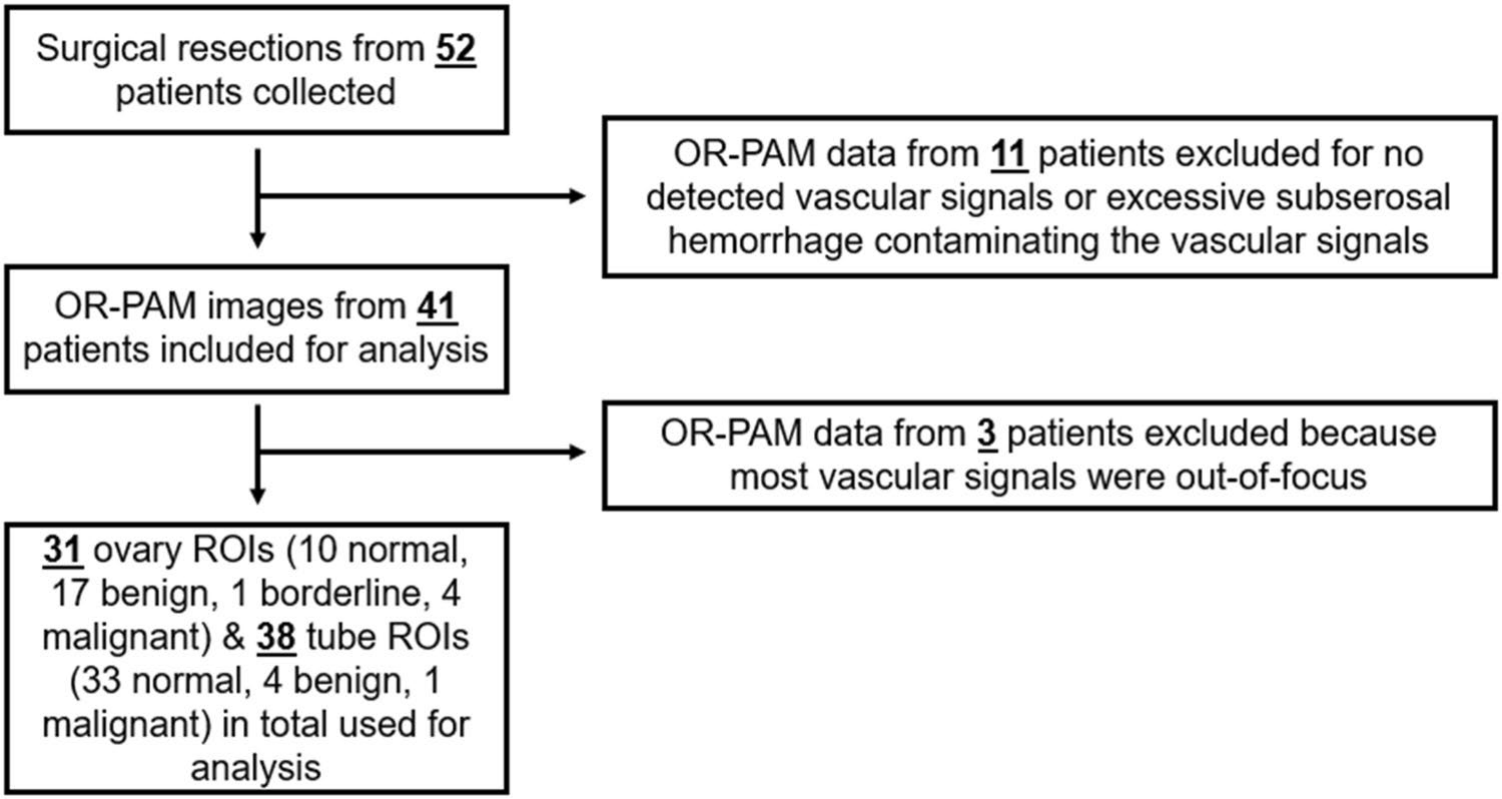
The data exclusion scheme and the total amount of data used for vasculature characterization.

An analysis pipeline was developed to characterize the vasculature of ovarian lesions and fallopian tubes. Datasets that contained significant contamination from subserosal hemorrhage or that had no blood vessel signals were excluded from further analysis. Additionally, images in which most of the vasculature was out-of-focus were also excluded. The data exclusion schema and the total amount of data used for analysis are illustrated in Fig. 10. Some patients contributed both an ovarian lesion and fallopian tube image, and more than one ROI from their specimens. In total, 31 ovarian lesion ROIs and 38 fallopian tube ROIs were used for vasculature characterization.

First, to reject out-of-focus signals, each C scan was time-gated for a depth range of 202.5 μm$\text{,}$ centered at the ultrasound focus. The variance of PA signals on the normalized data was calculated. Then this time-gated C scan was equally divided into 9 depth-resolved slabs, each 22.5 μm thick. Maximum intensity projection was calculated for each slab to obtain the vascular maps at 9 different depths. with a spacing of 22.5 μm. Lucy-Richardson deconvolution was applied to correct for the blood vessels in the first and last few slabs, which were expected to appear thicker because they were out of focus. For each vascular map, blood vessels were segmented as follows: the image was first contrast-enhanced using adaptive histogram equalization and binarized from a threshold found iteratively, based on the intensity range of the PA signals. To obtain a more accurate binary mask of the vasculature, small and isolated bleeding spots and vessel branches were removed and the vessels which appeared broken in the middle during binarization were linked. The vascular density was defined as the volume inside the 3D vascular mask divided by the total imaged volume. A built-in thinning algorithm in MATLAB was applied to each binary mask to extrapolate the centerlines of the segmented blood vessels, from which the total length of blood vessels in the imaged region was inferred by summing the lengths of all the centerlines. The mean vessel diameter was estimated as $\frac{\pi }{4}{D}^{2}=\frac{Vascular\, volume}{Total\, length\, of\, vessels}$, where the vascular volume was computed as the pixel count inside the vascular segmentation. A compensation factor of $\frac{2}{1+\sqrt{2}}$ was included to account for the underestimation of the lengths of diagonally oriented vessels. This estimation scheme was evaluated by imaging carbon fibers with an average diameter of 7 μm. Carbon fiber was chosen because its diameter approached the resolution limit of the system and could establish the smallest mean diameter for which this estimation scheme was applicable.

**Figure 11:**
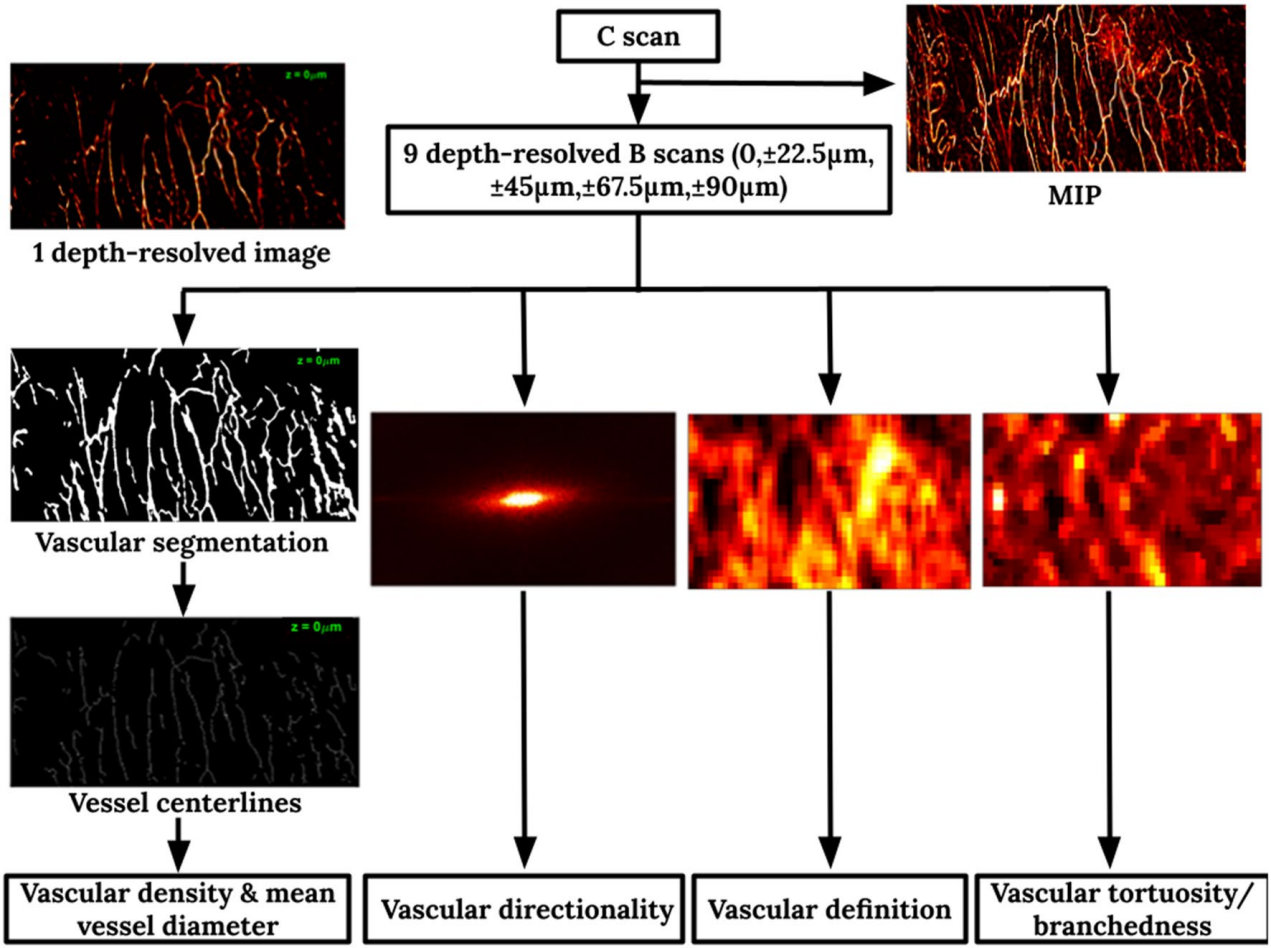
Processing flowchart for extracting vascular features from OR-PAM data.

Three additional vascular features were extracted, namely, the vascular directionality, the vascular definition, and the mean local tortuosity or branchedness. The vascular directionality was obtained by fitting an ellipse to the Fourier transform of the vascular mask and calculating its eccentricity. An eccentricity closer to 1 would indicate that the vessels in the imaged region were more directional, while an eccentricity closer to 0 would suggest that the vessels extended in all directions. The vascular definition was quantified indirectly from the local entropy of the images. Tiles with a width of 225 μm and 50% overlap with neighboring tiles were extracted from each image, and the entropy of each tile was computed. The mean and maximum entropies from all subregions in the image were found. A smaller entropy suggested a better definition of the local vasculature. In contrast, a larger/poorer definition of the vasculature was hypothesized to result from subserosal hemorrhage or irregular angiogenesis. Further, the local directionality indicated by the largest singular value (SV_0_) of each subregion was computed. By rotating the subregion $360^\circ$ in increments of $30^\circ$ and observing the variation of SV_0_ at different angles, the local directionality contrast was calculated. A small local directionality contrast could result from either high vessel tortuosity or more vessel branches. The mean and maximum local tortuosity/ branchedness values among all subregions were recorded from each image for further analysis. The processing pipeline is summarized in Fig. 11. The proposed feature extraction pipeline took about 15 min for each C scan.

Histopathologically normal ovary and normal fallopian tube samples were compared based on the extracted morphological features described above. A two-sample two-sided t-test was conducted to determine the features’ statistical significance in differentiating ovarian vasculature from fallopian tube vasculature. Additionally, benign (including cystic and fibromatous) and malignant tumors were characterized and compared to normal specimens.

Finally, some specimens were obtained from patients with known germline genetic mutations (e.g. *BRCA1*, *BRCA2*, *RAD51*) who were undergoing prophylactic salpingo-oophorectomy for risk reduction. This subset of patients is referred to as high-risk patients. These specimens, although manifesting no histopathological abnormalities at the time of the surgical procedure, were separated into a high-risk category, and their vascular characteristics were compared with those of normal specimens without genetic mutations.

### Data analysis

All analysis was performed using MATLAB R2019b. All methods were carried out in accordance with relevant guidelines and regulations**.**

## Data Availability

The data that support the plots within this paper and other findings of this study are available from the corresponding author upon request.
